# In vivo atherosclerotic plaque characterization using 7T quantitative T2*mapping distinguishes symptomatic middle cerebral artery plaques

**DOI:** 10.1016/j.jocmr.2026.102738

**Published:** 2026-04-30

**Authors:** Xiaoyan Bai, Ziming Xu, Tong Chen, Zhiye Li, Yi Ju, Xingquan Zhao, Qingle Kong, Zhe Zhang, Xue Zhang, Xun Pei, Yuanbin Zhao, Yajie Wang, Jiaqi Dou, Binbin Sui, Huijun Chen

**Affiliations:** a Department of Radiology, Beijing Friendship Hospital, Capital Medical University, Beijing, China; b Tiantan Neuroimaging Center for Excellence, China National Clinical Research Center for Neurological Diseases, Beijing Tiantan Hospital, Capital Medical University, Beijing, China; c Center for Biomedical Imaging Research, School of Biomedical Engineering, Tsinghua University, Beijing, China; d Department of Radiology, Beijing Tiantan Hospital, Capital Medical University, Beijing, China; e Department of Neurology, Beijing Tiantan Hospital, Capital Medical University, Beijing, China; f Department of Radiology, University of Southern California, Los Angeles, California, USA; g Department of Radiology, Beijing Hospital, Beijing, China

**Keywords:** Ultrahigh field magnetic resonance imaging, T2*mapping, Middle cerebral artery, Symptomatic plaque, Vessel-wall imaging

## Abstract

**Background:**

Intraplaque T2* values help to identify symptomatic carotid plaques and correlate with intraplaque iron deposits in plaque progression. However, intracranial T2* mapping in vivo at 3T magnetic resonance imaging (MRI) is challenging due to limited resolution and signal-to-noise ratio. This study aimed to quantitatively measure T2* value of middle cerebral artery (MCA) atherosclerotic plaques using 7T MRI and to assess its correlation with cerebrovascular symptoms.

**Methods:**

Phantom studies were performed to evaluate the accuracy of T2* mapping obtained with the proposed sequence by comparison with the ground truth acquisition. In the in vivo study, intraplaque T2* values obtained from multi-echo T2* mapping and plaque characteristics from T1-weighted three-dimensional (3D) sampling perfection with application-optimized contrast using different flip angle evolutions sequence on 7T MRI were analyzed and compared between patients with symptomatic and asymptomatic MCA plaques. Multivariate logistic regression was used to determine the odds ratios of T2* values and plaque characteristics in discriminating symptomatic from asymptomatic plaques. Diagnostic performance was evaluated using area under the curve (AUC) values. Correlation analyses were performed between T2* values and intraplaque hemorrhage (IPH).

**Results:**

Phantom T2* measurements using the proposed sequence showed excellent agreement with the ground truth sequence (ICC=0.998, p<0.001), with a mean percentage error of 3.97 ± 3.11%. The clinical cohort of this prospective cross-sectional study included 39 symptomatic patients with MCA plaques and 21 age-, sex-, and stenosis degree-matched asymptomatic patients. Scan–rescan reproducibility of T2* mapping was excellent (p<0.001). Symptomatic plaques had significantly lower T2* values than asymptomatic plaques (22.24±5.31 vs. 30.24±7.00 ms, p<0.001). In multivariate analysis, intraplaque T2* values (OR: 0.162, 95% CI: 0.053–0.497, p=0.001) and normalized wall index (NWI) (OR: 2.150, 95% CI: 1.041–4.443, p=0.039) were independently associated with symptomatic plaques. The optimal combination of T2* values and NWI showed the best diagnostic performance (AUC=0.861, 95% CI:0.747–0.937), with 94.9% sensitivity and 66.7% specificity. T2* values were negatively correlated with and IPH (r=−0.290, p=0.027) after age- and sex- adjustments.

**Discussion:**

The feasibility of intracranial T2*mapping in vivo on 7T MRI has been proven, indicating its potential as a sensitive tool for characterizing intracranial symptomatic plaques.

## Graphical abstract

**Figure:**
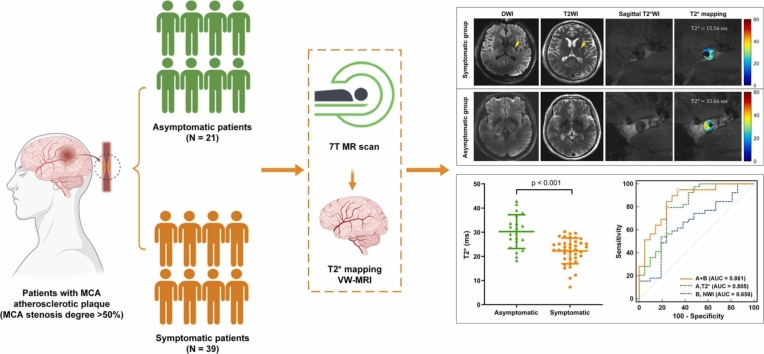

## 1 Introduction

Intracranial atherosclerotic plaques are a leading cause of ischemic stroke in Asian populations [1], [2], [3]. Patients with symptomatic plaques exhibit an increased risk of stroke recurrence, underscoring the importance of identifying symptomatic plaques [4]. High-resolution vessel wall magnetic resonance imaging (VW-MRI) has gained wide recognition as a potential noninvasive vascular imaging modality for evaluating intracranial plaque characteristics [5]. Recent meta-analyses on VW-MRI have revealed that intracranial plaque enhancement and T1 hyperintensity are robust imaging biomarkers for symptomatic plaques in patients with ischemic events [6], [7]. However, these qualitative assessments of imaging biomarkers rely on the signal intensity of surrounding tissues and are inherently subjective.

Quantitative MRI techniques are being developed and refined to characterize the vulnerability of carotid plaques [8], [9], [10]. By directly assessing the intrinsic quantitative values (T1, T2, and T2*), these techniques enable distinguishing plaque components independently of the signal intensity of the surrounding tissue, providing a new potential imaging index for plaque evaluation [10]. Specifically, T2* mapping allows quantitatively assessing iron deposition within carotid plaques and distinguishing symptomatic from asymptomatic plaques [11]. However, because intracranial arteries are small, and conventional MRI spatial resolution at 3T is limited, previous quantitative studies of intracranial plaques were typically conducted ex vivo [12].

With the development of physical MRI technology, 7T MRI has made huge breakthroughs in imaging resolution, offering improved spatial resolution, signal-to-noise ratios, and sensitivity of susceptibility [13]. The 7T MRI has been used to assess intracranial atherosclerotic disease [14], [15], [16], showcasing its potential for quantitatively assessing intracranial plaque characteristics. However, to date, no reports have been published on in vivo quantitative T2* mapping of intracranial plaques using 7T MRI. In this study, we hypothesized that 7T MR T2* mapping could be used to identify and assess the quantitative characteristics of intracranial atherosclerotic plaques in vivo.

This study was conducted to verify the feasibility of quantitatively measuring the T2* of intracranial atherosclerotic plaques in vivo using 7T MRI, and assess the predictive performance of intraplaque T2* values in differentiating symptomatic from asymptomatic plaques.

## 2 Methods

### 2.1 Patient population

This case-control study was conducted in accordance with the Declaration of Helsinki (as revised in 2013) and supported by the Institutional Review Board of Beijing Tiantan Hospital, Capital Medical University, China. All study participants provided written informed consent. From June 2021 to March 2023, symptomatic patients were considered for inclusion if (a) patients had an ischemic stroke [17] in MCA territory confirmed by diffusion-weighted imaging or clinical evidence of transient ischemic attack (TIA) [18] with ischemic symptoms corresponding to the MCA vascular distribution within the proceeding four weeks; (b) patients exhibited MCA stenosis >50% diagnosed by DSA, CTA, or MRA; (c) the M1 segment of the MCA atherosclerotic plaque was confirmed by VW-MRI; and (d) patients exhibited two or more vascular risk factors (hypertension, hyperlipidemia, diabetes mellitus, smoking, or coronary heart disease) [16]. Asymptomatic patients were included in this study if (a) the patients had no history of stroke or TIA but had MCA stenosis >50% confirmed by DSA, CTA, or MRA [19], [20], [21]; (b) the presence of an atherosclerotic plaque in the M1 segment of the MCA confirmed by VW-MRI, using the same imaging criteria applied to symptomatic patients; (c) the patients had two or more vascular risk factors; and (d) the age, sex, and degree of MCA stenosis of the asymptomatic patients were frequency-matched with those of the symptomatic patients. Age matching was performed within a comparable age range to ensure demographic comparability between groups. The sex distribution was closely matched between the two groups. In addition, the degree of MCA stenosis was matched using the same imaging modalities and diagnostic threshold (>50% stenosis) applied to symptomatic patients. Only asymptomatic subjects with a comparable severity of MCA stenosis were included. Exclusion criteria for all patients were (a) MCA occlusion; (b) the possibility that other factors, including cardioembolism (e.g., atrial fibrillation and infective endocarditis), aortic arch atheroma, or MCA lesions with ipsilateral carotid artery stenosis >50%, caused the stroke/TIA symptoms; (c) patients exhibited non-atherosclerotic vasculopathy, (e.g., vasculitis, dissection, vasospasm, or moyamoya disease); and (d) patients had claustrophobia or contraindications to MRI. Clinical and demographic data were recorded for all patients.

### 2.2 Magnetic resonance protocol

All scans were performed on a 7T MR scanner (MAGNETOM Terra, Siemens Healthineers, Erlangen, Germany) using a 32-channel Rx/8-channel Tx head-coil (Nova Medical, Wilmington, Massachusetts).

### 2.3 Phantom study

The phantom study was performed with 12 tubes filled with different concentrations of agarose and gadolinium-diethylenetriamine pentaacetic acid to create different T2* values of 5–60 ms. T2* was quantified using the multi-echo gradient-echo sequence with the following scan parameters: TR: 49 ms, 6-echo with TE: 4.7, 14.0, 19.1, 24.2, and 29.3 ms, flip angle: 15°, FOV: 200×200 mm^2^, slice thickness: 2 mm, number of slices: 1, in-plane resolution: 0.15×0.15 mm^2^, and scanning time: 3 min 14 s.

Apart from the proposed sequence for T2* quantification, the gradient-echo sequence with 24 different TE ranging from 4.7 to 34.4 ms (increasing by 1.3 ms) was performed as the ground truth of T2* mapping. After the first scan, the phantom was removed from the scanner and repositioned for a second scan. The localization and scan parameters of the first-second scans remain consistent.

### 2.4 In vivo study

The T1-weighted 3D sampling perfection with application-optimized contrast using different flip angle evolutions (SPACE) sequence parameters were as follows: repetition time (TR): 1720 ms, echo time (TE): 9.6 ms, field of view (FOV): 180×180×140 mm^3^, slice thickness: 0.40 mm, number of slices: 352, in-plane resolution: 0.40×0.40 mm^2^, the controlled aliasing in parallel imaging results in higher acceleration (CAIPIRINHA) factor: 6 (slice=2; phase=3), and scanning time: 9min 59s. VW-MR images obtained from the 3D T1 SPACE sequence were then transferred to a 3D viewer to localize a plane demonstrating the maximum MCA plaque (i.e., the plaque causing the most severe stenosis); this slice location was then used to localize the T2* measurement. T2* was quantified using the proposed 6-echo gradient-echo sequence same as the phantom study. Saturation bands were used for black blood vessel wall imaging.

### 2.5 Image analysis

For the phantom study, regions of interest (ROI) were automatically segmented on T2*-weighted images from the first echo using MATLAB (MathWorks, Natick, Massachusetts) to cover the entire tube area. T2* mapping was exponentially fitted by T2*-weighted images from 6 TEs and 24 TEs pixel by pixel, respectively.

**Fig. 1:**
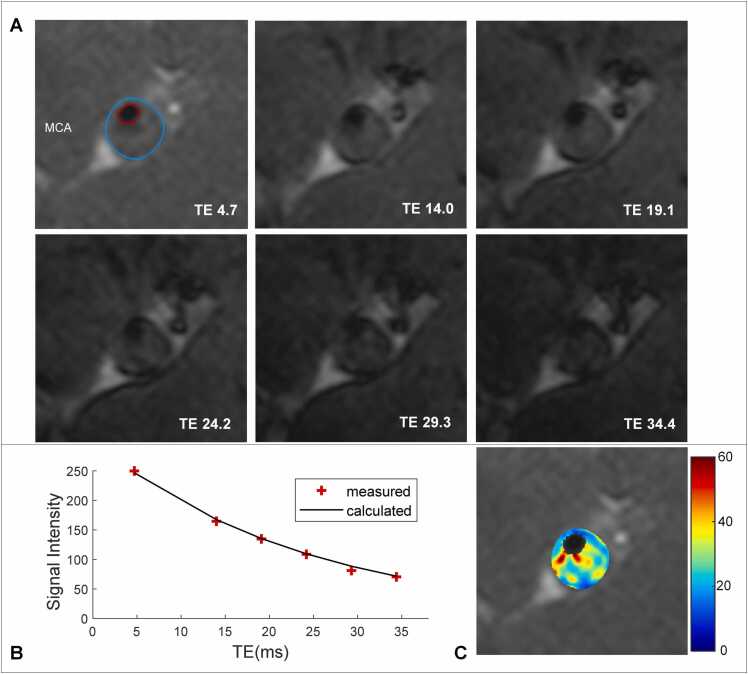
T2* weighted images, plaque signals, and intraplaque T2* mapping. (**A**) T2* weighted images of a symptomatic patient at various TEs: TE 4.7 ms; TE 14.0 ms; TE 19.1 ms; TE 24.2 ms; TE 29.3 ms; TE 34.3 ms. (**B**) Signal intensity curve was fitted by measured signals at each TE to calculate T2*. (**C**) Intraplaque T2* mapping. *MCA* middle cerebral artery, *TEs* echo times

For the in vivo study, the maximum MCA plaque was analyzed for both T2* mapping and vessel wall imaging. The vascular images were interpreted by two experienced reviewers (X.Z., with 5 years’ experience, and B.X., with 8 years’ experience) who were blinded to clinical information and brain images. The regions between the outer wall and lumen of the selected plaques were regarded as regions of interest (ROIs) and were manually delineated on T2*-weighted images using our in-house software. T2* mapping was exponentially fitted pixel by pixel by T2*-weighted images from six TEs [11], and the mean T2* value of each ROI was recorded. Fig. 1 shows the T2*-weighted images and T2* mapping of one representative symptomatic patient. The MCA stenosis degree and plaque characteristics, including maximum wall thickness (Max WT), normalized wall index (NWI), and intraplaque hemorrhage (IPH), were defined and analyzed as described previously [16].

### 2.6 Reproducibility and accuracy assessment

The T2* quantification accuracy of the proposed method was evaluated by scanning phantoms and comparing them with the ground truth acquisition.

Ten patients with MCA atherosclerotic stenosis who met the inclusion criteria were randomly selected for reproducibility tests. The scan–rescan reproducibility of plaque T2* quantification was assessed by measuring the agreement between independent 6-echo acquisitions (proposed sequences) in patients with MCA atherosclerotic plaque. After the first scan, patients were temporarily removed from the scanner and asked to leave the MRI examination area. They returned to the scanner room after a 20-minute interval to undergo the second scan. The imaging localization and scanning parameters of the two acquisitions were kept consistent.

Twenty patients were randomly selected for testing the inter-observer reproducibility in identifying the presence of IPH and measuring NWI, Max WT, and T2* value at MCA.

### 2.7 Statistical analysis

The statistical analysis was performed using SPSS 26.0 software (SPSS Inc., Chicago, Illinois, USA) and R software (V 1.0, The R Foundation for Statistical Computing, Vienna, Austria), and p<0.05 was considered statistically significant. Phantom T2* measurements obtained with the proposed sequence were compared with those derived from the ground truth sequence using the intraclass correlation coefficient (ICC). Bland–Altman analysis was additionally performed to evaluate measurement agreement and to assess potential systematic bias between the two methods. Scan–rescan tests difference for the proposed sequence was also assessed using ICC, with Bland–Altman plots further applied to assess scan–rescan repeatability and agreement.

For the in vivo study, all quantitative data are expressed as means ± standard deviations or median (range). Categorical variables were analyzed using chi-square or Fisher’s exact tests. Continuous variables were compared using independent *t*-tests, the Mann–Whitney *U* test or one-way analysis of variance. Univariable and multivariable logistic regression analyses were performed to evaluate clinical and plaque characteristics in discriminating symptomatic from asymptomatic plaques. The univariate variables with p<0.10 were further input into the multivariate logistic analysis with a backward selection to identify the independent predictive factors associated with the symptomatic plaque. The predictive performance was described using receiver-operating-characteristic (ROC) curves and area under the curve (AUC) values. Comparisons of ROC curves were performed using the method developed by DeLong et al. [22]. To further evaluate the incremental clinical value of the combined model over the single-parameter model, reclassification analyses were performed. Specifically, the net reclassification improvement (NRI) was calculated to quantify the extent to which the combined model improved patient risk stratification compared with the single-parameter model. Correlations between intraplaque T2* values and IPH were determined using partial correlation analysis with age and sex as covariates. The ICC and Bland–Altman analyses were used to assess repeatability and agreement between scan–rescan tests. The inter-observer reproducibility of continuous variables was evaluated using ICCs, while Cohen’s kappa was determined for the categorical variables.

## 3 Results

### 3.1 Phantom study

**Fig. 2:**
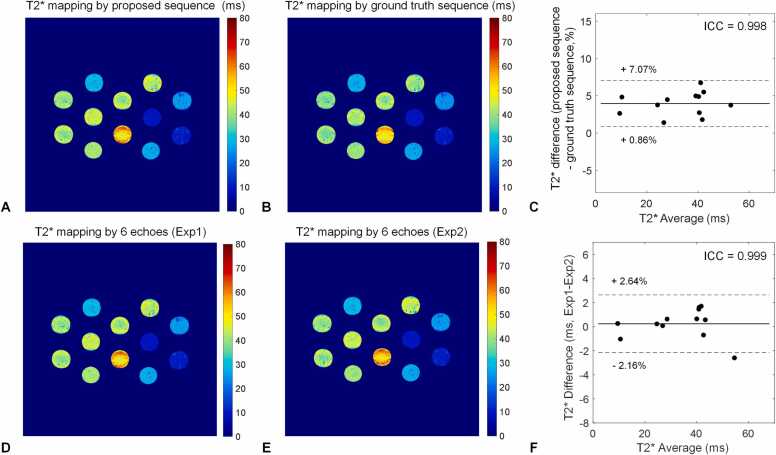
Accuracy and reproducibility of T2* mapping in the phantom study. T2* mapping was estimated using GRE sequence with the proposed sequence (**A**) and ground truth sequence (**B**). Bland–Altman plot illustrates the differences between the two methods, with the solid line representing the mean difference (3.97%) and the dashed lines indicating the 95% limits of agreement (0.86% to 7.07%) (**C**). T2* mapping derived from the proposed GRE sequence for the first (**D**) and second scans (**E**) are shown. Bland–Altman analysis of the scan–rescan measurements displays the mean difference (0.24%) and the 95% limits of agreement (−2.16% to 2.64%) (**F**). *GRE* gradient recalled echo

The accuracy of T2* mapping was validated by comparison between the proposed sequence and ground truth sequence. Fig. 2 A–B showed T2* mapping of phantoms generated from the proposed sequence and ground truth. T2* values estimated with the two methods showed excellent correlation (ICC=0.998, 95% CI 0.993–0.999, p<0.001). The Bland–Altman plot showed that the mean percentage error in T2* measurement using the proposed sequence imaging was 3.97± 3.11% compared to the ground truth sequence (Fig. 2C). Fig. 2 D–E demonstrated T2* mapping of phantoms generated with the proposed sequence from two independent scans. The Bland–Altman plot showed the differences between the two scans were 0.24±2.40% (Fig. 2F). Excellent agreement between the scan–rescan tests can be observed in measuring T2* values (ICC=0.999, 95% CI 0.997–1.000, p<0.001).

### 3.2 In vivo study

#### 3.2.1 Patient demographics

From June 2021 to March 2023, 45 symptomatic patients with MCA stenosis >50% were recruited. Six patients were excluded owing to ipsilateral carotid artery stenosis >50% (n=1), vasculitis (n=1), dissection (n=1), moyamoya disease (n=1), and claustrophobia (n=2). Thirty-nine symptomatic patients (27 males; mean age: 49.79±13.42 years; MCA stenosis degree: 84.52±10.90) were included. Twenty-one asymptomatic patients matched for age-, sex-, and stenosis degree (15 males; mean age: 48.52±12.34 years; MCA stenosis degree: 81.25±11.38) were also included in the final study. Table 1 list the patient’s demographic features. Age, sex, MCA stenosis degree, clinical risk factors, and blood test results did not significantly differ between symptomatic and asymptomatic patients (all p>0.05).

**Table 1:** Baseline demographics and MCA plaque characteristics between asymptomatic and symptomatic patients

	Asymptomatic (n = 21)	Symptomatic (n = 39)	*p* value
Age (y)	48.52±12.34	49.79±13.42	0.720
Male, n (%)	15 (71.4)	27 (69.2)	0.859
Stenosis degree of MCA (%)	81.25±11.38	84.52±10.90	0.280
Clinical risk factors			
Smoking, n (%)	11 (52.4)	18 (46.2)	0.645
Drinking, n (%)	10 (47.6)	19 (48.7)	0.935
Hypertension, n (%)	13 (61.9)	25 (64.1)	0.866
Diabetes mellitus, n (%)	7 (33.3)	9 (23.1)	0.392
Hyperlipidemia, n (%)	8 (38.1)	16 (41.0)	0.825
Coronary heart disease, n (%)	2 (9.5)	3 (7.7)	>0.999
Laboratory test			
Total cholesterol, TC (mmol/L)	3.84±0.83	4.01±0.80	0.486
Triglyceride, TG (mmol/L)	1.54±0.58	1.93±0.88	0.102
High-density lipoprotein, HDL (mmol/L)	1.20±0.31	1.12±0.23	0.290
Low-density lipoprotein, LDL (mmol/L)	2.05±0.61	2.21±0.73	0.418
High-sensitive C-reactive protein, hsCRP (mg/L)	0.38 (0.27-1.23)	1.27 (0.39-2.49)	0.178
Homocysteine, HCY (µmol/L)	11.57 (9.80-14.10)	13.58 (10.40-20.01)	0.158
Neutrophil-to-lymphocyte ratio, NLR	2.36 (1.64-3.77)	2.25 (1.78-3.31)	0.924
Lymphocyte-to-monocyte ratio, LMR	4.52 (3.14-5.90)	5.09 (4.22-6.16)	0.322
Acute/Subacute infarction, n (%)	-	36 (92.3)	-
Onset to MRI time, (d)	-	14.50 (5.00-30.00)	-
MCA plaque characteristics			
NWI	0.89 (0.81-0.93)	0.93 (0.88-0.95)	0.048*
Max WT (mm)	1.24 (1.07-1.77)	1.50 (1.14-1.82)	0.329
IPH, n (%)	0 (0.0)	6 (15.4)	-
T2* (ms)	30.24±7.00	22.24±5.31	<0.001*

Notes: Continuous variables were expressed as mean ± SD for the normal distribution data and median with interquartile range for the non-normal distribution data; Categorical variables were expressed as percentages. *MCA* Middle cerebral artery, *NWI* Normalized wall index, *Max WT* Maximal wall thickness, *IPH* Intraplaque hemorrhage. *p<0.05 was recognized as statistically different

#### 3.2.2 Plaque characteristics between symptomatic and asymptomatic plaques

**Fig. 3:**
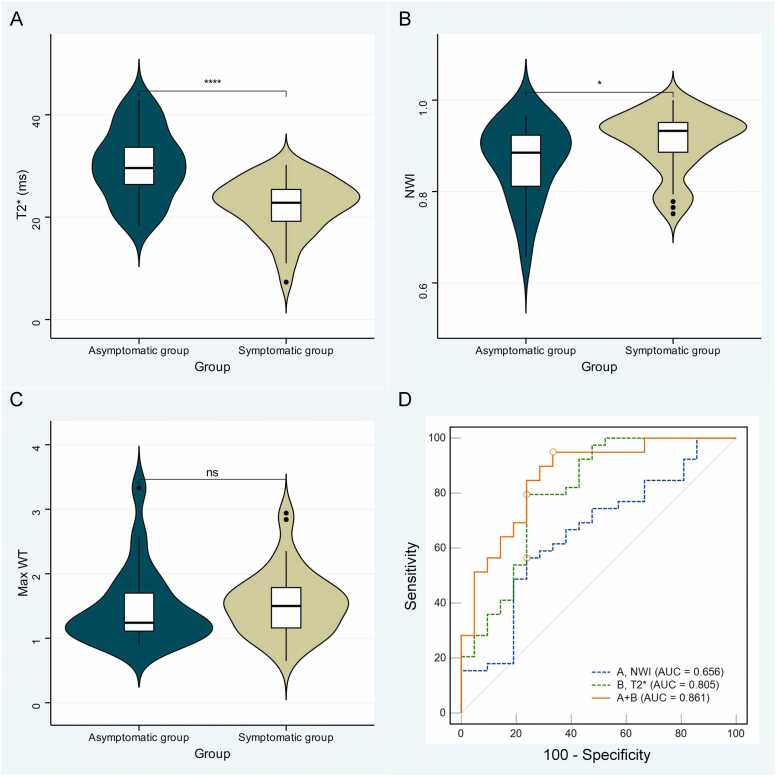
Comparison of T2* values between asymptomatic and symptomatic plaques. Symptomatic group showed significantly lower mean intraplaque T2* values (**A**) and NWI (**B**) than asymptomatic group. No significant difference was found in the Max WT (**C**) between the two groups. ROC curves showed that the optimal combination of intraplaque T2* value and NWI could improve the predictive performance in identifying symptomatic plaques. *, indicates a statistical significance (*p* < 0.05); ***, indicates a statistical significance (p<0.001); ns, indicates no statistical significance. *ROC* receiver operating characteristic, *Max WT* maximum wall thickness, *NWI* normalized wall index

**Fig. 4:**
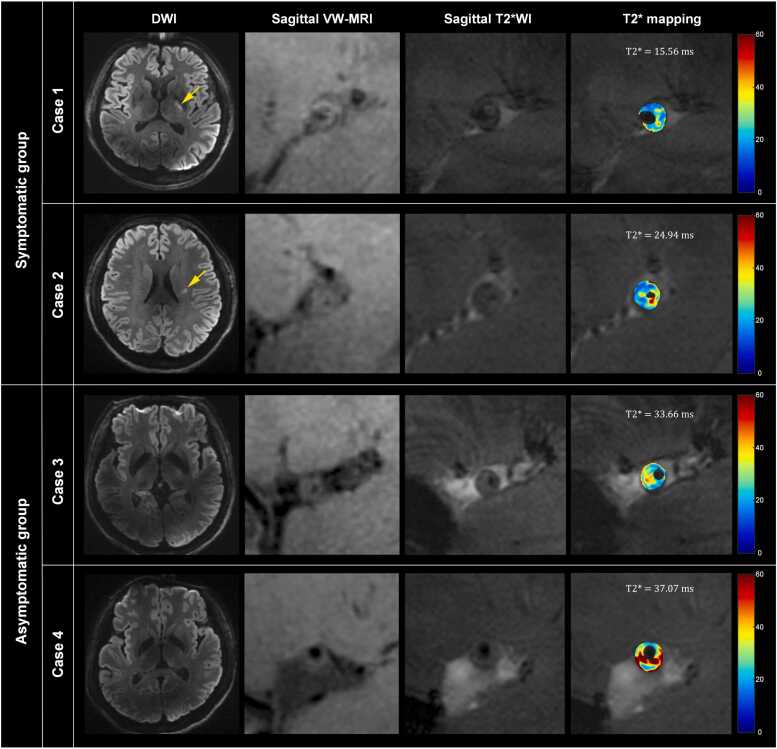
Representative quantitative MRI images in symptomatic and asymptomatic patients. Four cases are presented, illustrating DWI, sagittal VW-MRI, sagittal T2*WI, and T2* mapping. In the symptomatic group, DWI revealed hyperintense lesions in the left basal ganglia territory (case 1) and the left corona radiata (case 2) (yellow arrows). No infarction was detected on DWI in the asymptomatic group (cases 3 and 4). Sagittal VW-MRI and T2*WI images display MCA plaques, with corresponding mean T2* values derived from T2* mapping. *DWI* diffusion-weighted imaging, *VW-MRI* vessel wall magnetic resonance imaging, *MCA* middle cerebral artery

The symptomatic group exhibited a significantly lower intraplaque T2* value than the asymptomatic group (22.24±5.31 vs. 30.24±7.00 ms, p<0.001; Table 1). Additionally, the symptomatic group had significantly larger NWI value than the asymptomatic group (p=0.048). Max WT did not significantly differ between the two groups (p=0.329; Fig. 3). Six patients in the symptomatic group had IPH (15.4%, 6/39), whereas no patients in the asymptomatic group had IPH (0.0%, 0/21). Fig. 4 shows four representative MR imaging from symptomatic and asymptomatic patients.

#### 3.2.3 Clinical and plaque characteristics for differentiating symptomatic from asymptomatic plaques

Univariable logistic regression analysis identified NWI (OR: 1.767, 95% CI: 1.010–3.092, p=0.046) and intraplaque T2* value (OR: 0.173, 95% CI: 0.064–0.469, p=0.001) as inputs for the multivariable analysis (Table 2). Multivariable logistic regression analysis showed that NWI (OR: 2.150, 95% CI: 1.041–4.443, p=0.039) and intraplaque T2* value (OR: 0.162, 95% CI: 0.053–0.497, p=0.001) were independently associated with symptomatic plaques.

**Table 2:** Univariate and multivariate logistic analyses for symptomatic plaques

Characteristics	Univariate regression analysis			Multivariate regression analysis		
	OR	95% CI	*p* value	OR	95% CI	*p* value
Age	1.008	0.97–1.050	0.715			
Male	1.111	0.346–3.564	0.859			
Smoking	0.779	0.269–2.256	0.646			
Drinking	1.045	0.361–3.022	0.935			
Hypertension	1.099	0.367–3.292	0.866			
Diabetes mellitus	0.600	0.185–1.941	0.394			
Hyperlipidemia	1.130	0.381–3.354	0.825			
Coronary heart disease	0.792	0.122–5.155	0.807			
Total cholesterol	1.241	0.683–2.255	0.479			
Triglyceride	1.769	0.881–3.548	0.109			
High-density lipoprotein	0.730	0.408–1.304	0.287			
Low-density lipoprotein	1.299	0.695–2.427	0.412			
hsCRP	3.003	0.474–19.025	0.243			
Homocysteine	1.087	0.981–1.204	0.112			
NLR	0.962	0.667–1.389	0.838			
NWI	1.767	1.010–3.092	0.046*	2.150	1.041–4.443	0.039*
Max WT	1.134	0.654–1.968	0.655			
T2* value	0.173	0.064–0.469	0.001*	0.162	0.053–0.497	0.001*

Notes: *MCA* Middle cerebral artery, *hsCRP* High-sensitive C-reactive protein, *NLR* Neutrophil-to-lymphocyte ratio, *NWI* normalized wall index, *Max WT* maximal wall thickness, *OR* odds ratio, *CI* confidence interval. *p<0.05 was recognized as statistically different

ROC analysis indicated that using the cut-off value of 26.23 ms, intraplaque T2* value showed the best performance (AUC=0.805, 95% CI 0.680–0.929, p=0.001) for differentiating symptomatic from asymptomatic plaques, with 79.5% sensitivity, and 76.2% specificity. NWI showed the predictive performance (AUC=0.656, 95% CI: 0.509–0.802, p=0.048) to identify symptomatic plaques with 56.4% sensitivity, and 76.2% specificity. The optimal combination of intraplaque T2* value and NWI had an AUC of 0.861 (95% CI: 0.747–0.937), which significantly improved the predictive performance of symptomatic plaques with 94.9% sensitivity, and 66.7% specificity (Fig. 4). Comparing the ROC curves showed that the AUC of the model incorporating intraplaque T2* value and NWI was significantly higher than that of single model using NWI alone (AUC= 0.861 vs. 0.656; p=0.015). No significant difference in AUC was observed between the combined model and the single model using T2* value alone (p=0.152), nor between the single T2*-based model and the NWI-based model (p=0.175). Compared with the single model using T2* value, the combined model achieved a continuous NRI of 0.73 (95% CI: 0.20–1.23, p=0.010).

#### 3.2.4 Correlation between intraplaque T2* values and IPH

**Fig. 5:**
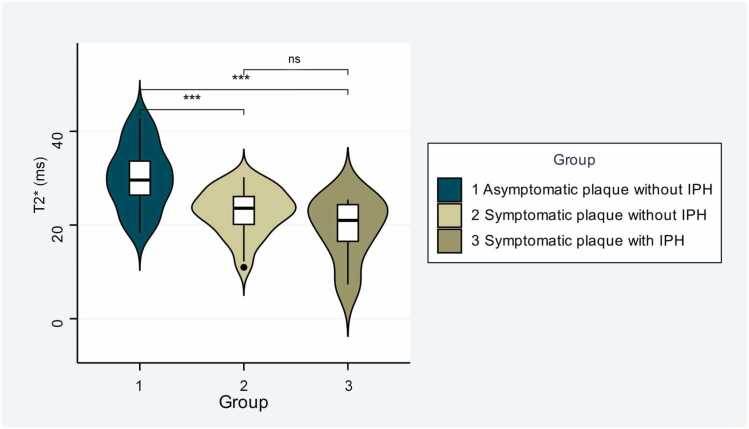
Comparisons of T2* values among different subgroups in symptomatic and asymptomatic plaques. Intraplaque T2* values in symptomatic plaques without and with IPH were significantly lower than those in asymptomatic plaques without IPH. ***, indicates a statistical significance (p<0.001); ns, indicates no statistical significance. *IPH* intraplaque hemorrhage

Intraplaque T2* values were negatively correlated with IPH (r=−0.290, p=0.027, n=60) after age and sex adjustments. Compared with asymptomatic plaques without IPH, intraplaque T2* values were significantly decreased in symptomatic plaques without IPH (30.24±7.00 ms vs. 22.79±4.90 ms, p<0.001) and symptomatic plaques with IPH (30.24±7.00 ms vs. 19.20±6.90 ms, p<0.001; Fig. 5). No significant differences were found between symptomatic plaques without IPH and with IPH (22.79±4.90 ms vs. 19.20±6.90 ms, p=0.528).

#### 3.2.5 Reproducibility and accuracy assessment

**Fig. 6:**
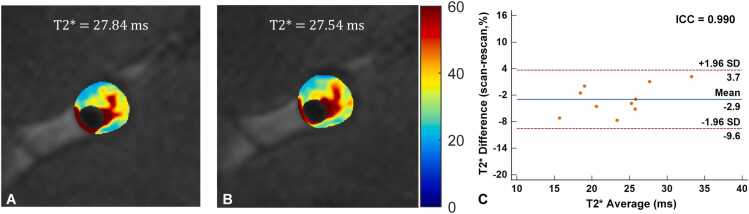
One representative case of T2* mapping from the repeatability scan. T2* mapping is presented for the first (**A**) and second scans (**B**). Bland–Altman plot showing the differences between the scan–rescan with the mean values (**C**). The solid line indicates the average difference (−2.9%), and the dotted lines indicate the 95% limits of agreement (−9.6% and 3.7%)

Ten patients (7 symptomatic and 3 asymptomatic) with MCA atherosclerotic stenosis underwent scan–rescan tests to assess the scan–rescan reproducibility of intraplaque T2* values. The scan–rescan tests showed excellent agreement for measuring T2* values (ICC=0.990, 95% CI: 0.955–0.997, p<0.001; Fig. 6).

For the inter-rater agreement in the assessment of presence of IPH, the kappa value was 0.77 (p<0.001). The inter-observer ICC was 0.947 (95% CI, 0.866–0.979), 0.872 (95% CI, 0.677–0.949), and 0.989 (95% CI, 0.972–0.996) for measuring NWI, Max WT, and T2* values, respectively.

## 4 Discussion

Our study demonstrated the significance of intraplaque T2* values as independent indicators for identifying symptomatic plaques. This underscore the potential of quantitative and noninvasive T2* mapping assessment as a novel and sensitive tool for distinguishing symptomatic from asymptomatic plaques. In our phantom study, we verified the accuracy of T2* quantification of the proposed method by comparing it with the ground truth acquisition. We introduced a feasible T2* mapping scanning protocol capable of accurately measuring T2* values within a reasonable time. Furthermore, in vivo studies showed consistent results in repeated measurements of T2* values. To our knowledge, this is the first report of in vivo T2* mapping of intracranial atherosclerotic plaques using 7T MR imaging.

In the present study, quantitative analysis of T2* mapping revealed that intraplaque T2* values in the symptomatic group are lower than those in the asymptomatic ones. Consistently, a previous MRI study revealed lower intraplaque T2* values in symptomatic carotid plaques compared with asymptomatic carotid plaques, and identified the dynamic presence of iron within the atherosclerotic carotid plaque microenvironment ex vivo [11]. These observations suggest that T2* is sensitive to biologically relevant differences in intraplaque iron chemistry and aggregation state. Mechanistically, T2* shortening has been linked to a shift toward aggregated iron complexes that generate stronger local magnetic susceptibility effects, even when total iron content is similar. This highlights the unique sensitivity of T2* to iron species and microstructural organization within plaques. Supporting this interpretation, histopathological studies reported that symptomatic carotid plaques had higher iron concentrations, signs of cap rupture, and increased cap macrophage activity compared with asymptomatic ones, indicating a positive correlation between iron deposition and plaque vulnerability in carotid arteries [23], [24]. Iron accumulation within atherosclerotic plaques may arise from multiple biological processes, including microhemorrhage, macrophage-mediated phagocytosis, and degradation of aging erythrocytes, all of which lead to redox-active iron accumulation [25], [26]. Paramagnetic iron-containing compounds such as ferritin and hemosiderin can substantially shorten T2* relaxation time, making T2* a sensitive imaging biomarker for iron accumulation [27], [28]. In this study, the decreased T2* values within symptomatic plaques may be primarily attributed to iron deposition. This leads to measurable changes in local magnetic field homogeneity and shortens T2* relaxation time. In our study, we used the advanced 7T MR technology to directly quantify intracranial T2* values in vivo, potentially providing an innovative approach for characterizing intracranial plaque types.

In addition to considering iron deposition, calcium presence may cause interference in measuring T2* values, as calcium can also shorten T2* relaxation time. A previous study by Baek et al [29]. discovered that calcification of MCA atherosclerotic lesions was rare, with a significantly lower incidence within symptomatic MCA atherosclerotic plaques (4.2%) than in asymptomatic plaques (7.9%). Nevertheless, the current study cannot rule out the effect of microcalcification deposits on intraplaque T2* values. Quantitative MRI studies on intracranial plaques have been conducted ex vivo, focusing on T1/T2/T2* mapping to distinguish different components (including lipid core and fibrous tissue) based on relaxation times, without addressing intracranial intraplaque iron or calcification deposition [12], [30]. Similar quantitative MRI studies on intracranial plaques in vivo have been rarely reported. Future histopathological studies should validate the presence of plaque calcification or microcalcifications.

Intraplaque hemorrhage serves as a robust biomarker for plaque vulnerability and stroke risk [31], [32]. Here, intraplaque T2* values were associated with the presence of IPH, indicating that lower T2* values are associated with an increased likelihood of visually detectable IPH. To further explore the potential influence of IPH on T2* measurements, we conducted a subgroup analysis based on the presence or absence of IPH. Interestingly, our study revealed that intraplaque T2* values were significantly lower in symptomatic plaques, both with and without visually detectable IPH when compared with asymptomatic plaques without IPH. In other words, even in the absence of visually detectable IPH, T2* values in the symptomatic group consistently remained lower than those in the asymptomatic group. These findings suggest that decreased T2* values may reflect pathological features beyond macroscopically visible IPH. One plausible explanation is the presence of microhemorrhages below the detection threshold of conventional qualitative VW-MR imaging. Qualitative IPH assessment based on conventional T1-weighted imaging has inherent limitations and may fail to identify subtle or early-stage intraplaque microhemorrhage. In this context, T2* mapping provides complementary information by offering quantitative, susceptibility-sensitive measurements that enable objective assessment of iron-related components within atherosclerotic MCA plaques. By reflecting local magnetic susceptibility variations associated with iron chemistry and microstructural organization, T2* mapping may improve sensitivity to subtle iron-related pathological changes that are not visually apparent on conventional T1-weighted images, especially at ultrahigh field strengths. In our study, we emphasized that T2* values may serve as a novel and more sensitive complementary parameter rather than a substitute for IPH assessment. Building on conventional VW-MR imaging, quantitative T2* mapping may improve the detection of iron-related pathological changes, including possible microhemorrhage, especially in patients without visually apparent IPH. Consequently, decreased intraplaque T2* values may suggest increased plaque vulnerability in patients with intracranial atherosclerosis, although further histopathological validation is required. Future studies, especially those with large sample sizes, are warranted to confirm these observations.

Plaque burden is another significant indicator for evaluating plaque vulnerability and progression in intracranial lesions [33]. Plaque tension increases with increasing plaque burden, which can aggravate the lumen stenosis and insufficient hemodynamic perfusion in the MCA territory. Despite similar degrees of MCA stenosis between symptomatic and asymptomatic groups in this study, NWI remained a crucial independent indicator for identifying symptomatic plaques. This provides compelling evidence that plaque burden may be a more effective indicator of symptomatic lesions than luminal stenosis, considering potential positive remodeling in atherosclerotic arteries [6]. Moreover, the combination of NWI and intraplaque T2* value provided complementary information and demonstrated greater discriminatory power than single-parameter alone for identifying symptomatic plaques. In this context, intraplaque T2* serves as an additional marker reflecting plaque composition, thereby enhancing risk stratification beyond structural burden alone. Consistent with this, reclassification analysis further demonstrated that, compared with the single model using T2* value, the combined model provided incremental clinical value by more appropriately reclassifying a substantial proportion of patients, despite only modest improvements in AUC. More specifically, among the 60 patients, 29 of 39 symptomatic patients were reclassified upward and 13 of 21 asymptomatic patients were reclassified downward, reflecting a net improvement in classification performance. Taken together, these findings indicate that the combined model incorporating intraplaque T2* value and NWI provides clinically meaningful incremental value through improved patient risk stratification. From a pathophysiological perspective, plaque burden represents the overall disease severity and mechanical stress environment [34], whereas intraplaque T2* reflects iron-related pathological processes such as microhemorrhage and macrophage activity [11]. Therefore, their combined use may enable a more comprehensive characterization of plaque vulnerability and improve the identification of high-risk intracranial plaques.

## 5 Limitations

This study had several limitations. First, it was a cross-sectional study with a relatively small sample size. To investigate MCA plaque characteristics associated with recurrent stroke, a longitudinal study with follow-up imaging is warranted. Second, to adequately compare of intraplaque T2* values between symptomatic and asymptomatic groups, we included patients from both groups matched for MCA stenosis degree. Thus, T2* values serve as a plaque characteristic that remains independent of the MCA stenosis degree. Third, histopathological validation of MCA plaque components was not available in the present study. Future studies must address the lack of histological validation by histopathologically analyzing the composition of intracranial atherosclerotic plaques to validate our findings. Additionally, owing to the two-dimensional acquisition of the quantitative T2* mapping sequence used herein, only one slice with maximum vessel wall thickness in the stenotic MCA was selected for vertical scanning and plaque T2* measurement, and this region might not have fully reflected the T2* value of the entire plaque. Volumetric assessment could be achieved with the development of three-dimensional T2* mapping techniques. In addition, owing to the substantially increased SAR at ultrahigh field strengths, safety constraints require prolonged repetition times, resulting in excessive scan durations and increased susceptibility to motion artifacts. Consequently, robust in vivo T1 and T2 mapping could not be feasibly implemented in the imaging protocol of the present study. Future advances in T1 and T2 mapping techniques with low-SAR and better B1⁺ correction may enable multiparametric characterization of intracranial plaque tissue at ultrahigh-field strengths, providing complementary information for plaque components characterization and high-risk plaque identification. Furthermore, the Bland–Altman analysis in this study was based on percentage differences, which was intentionally adopted to normalize measurements across the wide T2* range. However, this approach may obscure proportional bias, as the bias is not constant but varies proportionately with T2* values; therefore, the agreement results should be interpreted with this consideration in mind. Moreover, because the interval used for the interstudy repeatability assessment was relatively short, the potential influence of day-to-day variations on the results may not have been fully captured, which warrants further investigation in future studies. Finally, this study did not collect the serologic indicators of total body iron stores, which may provide epidemiologic evidence in early atherosclerotic atherosclerosis [35]. Future research should explore the relationship between serological indicators of body iron stores and T2* values.

## 6. Conclusions

The feasibility of quantitative T2* mapping of intracranial plaque can be demonstrated at 7T MRI. This quantitative and noninvasive technology may serve as a new and sensitive tool for characterizing intracranial symptomatic plaques.

## Funding

This study has received funding by Beijing Natural Science Foundation (Grant No. L232130, 7212028, 7162056, L252052, L242045, and L246019); 10.13039/501100001809National Natural Science Foundation of China (Grant No. 62271061, 81930119, and 82502288); National High Level Hospital Clinical Research Funding (BJ-2025–042, BJ-2025–132); Beijing Municipal Science & Technology Commision (Z231100004823012).

## Author contributions

X-YB and Z-MX planned the study, analyzed the data, interpreted the findings and wrote the manuscript; YJ and X-QZ enrolled patients; X-YB, TC, Z-YL, XZ, XP, and Y-BZ contributed to data collection; Z-MX, Y-JW, Q-LK, ZZ, and J-QD debugged image sequences; Z-MX, YJ, X-QZ, TC, Z-YL, XZ, XP, Y-BZ, H-JC, and B-BS provided critical comments/ revisions of the manuscript; H-JC and B-BS are responsible for the overall content.

## Ethics approval and consent

The study protocol was approved by the Institutional Review Board of Beijing Tiantan Hospital, Capital Medical University, and the written consent forms were obtained from all the subjects prior to the initiation of this study.

## Declaration of competing interests

The review authors have no competing interests to declare.

## Data Availability

The datasets generated during and/or analyzed during the current study are available from the corresponding author on reasonable request.
